# Remnant Cholesterol and Dyslipidemia Are Risk Factors for Guillain–Barré Syndrome and Severe Guillain–Barré Syndrome by Promoting Monocyte Activation

**DOI:** 10.3389/fimmu.2022.946825

**Published:** 2022-07-13

**Authors:** Yaowei Ding, Lijuan Wang, Jialu Sun, Yijun Shi, Guoge Li, Xin Luan, Guanghui Zheng, Guojun Zhang

**Affiliations:** ^1^ Department of Clinical Diagnosis, Laboratory of Beijing Tiantan Hospital, Capital Medical University, Beijing, China; ^2^ National Medical Products Administration (NMPA) Key Laboratory for Quality Control of In Vitro Diagnostics, Beijing Tiantan Hospital, Capital Medical University, Beijing, China; ^3^ Beijing Engineering Research Center of Immunological Reagents Clinical Research, Beijing Tiantan Hospital, Capital Medical University, Beijing, China

**Keywords:** Guillain-Barré syndrome (GBS), remnant cholesterol, dyslipidemia, monocyte activation, Guillain-Barré syndrome disability score (GBS-DS)

## Abstract

**Background:**

Guillain–Barré syndrome (GBS) is the most common severe acute paralytic neuropathy, with a mortality rate of 5% and permanent sequelae rate of 10%. Currently, the cause of GBS remains unclear. Therefore, we sought to determine potential predictors for GBS and its severity.

**Methods:**

A case–control study was performed at Tiantan Hospital in Beijing from January 2017 to December 2021. Laboratory and clinical characteristics were assessed in recruited GBS patients and healthy control individuals (matched by sex and age). The potential risk factors for GBS and severe GBS were assessed using a logistic regression analysis. The mRNA levels of toll-like receptor 4 (TLR4), toll-like receptor 2 (TLR2) and nuclear factor κB (NF-κB) in GBS patients and control PBMCs were detected by fluorescence quantitative PCR. THP-1 cells were costimulated with LPS and free cholesterol to demonstrate the effect of free cholesterol on monocyte activation.

**Results:**

A total of 147 GBS patients and 153 healthy individuals were included in the study. Logistic regression analyses showed that preceding infection, alcohol consumption, remnant cholesterol, homocysteine and the dyslipidemia index were correlated with a higher risk of GBS. In contrast, increased HDL cholesterol was correlated with a lower risk of GBS. Moreover, remnant cholesterol and the dyslipidemia index were significantly correlated with severe GBS. The mRNA levels of TLR4, TLR2 and NF-κB in the PBMCs of GBS patients were significantly higher than those of healthy individuals. LPS activated THP-1 cells, and free cholesterol treatment increased the expression of TLR4, TLR2, NF-κB and IL-1β mRNA in LPS-activated THP-1 cells.

**Conclusion:**

Dyslipidemia was correlated with the risk of GBS and severe GBS. Remnant cholesterol may promote the activation of monocytes in GBS patients. It may be valuable to control lipid levels in the prevention of GBS and severe GBS.

## Introduction

Guillain–Barré syndrome (GBS) is an autoimmune demyelinating disease in the peripheral nervous system (PNS). The pathological features are infiltration of inflammatory cells into small blood vessels and demyelination of peripheral nerves and nerve roots (1). The incidence of GBS is 0.8-1.9 (median 1.1) per 100,000 person-years and increases with age. The proportion of GBS patients is higher in males than in females (2, 3). The fatality rate is 5%, and permanent sequelae are observed in 10% of patients. GBS usually follows an abnormal autoimmune response to peripheral nerves and spinal cord roots caused by infection or other immune stimulation. Recent studies have shown that GBS is one of the serious complications of COVID-19 and one of the serious adverse reactions to COVID-19 vaccination (2, 4).

There have been many studies on the clinical epidemiology of GBS, but its cause is not completely clear. A proteomics study of the cerebrospinal fluid of GBS patients showed that the differentially expressed proteins between the GBS and control groups were mainly enriched in pathways closely related to lipid metabolism (5). It is interesting that the concept of cholesterol toxicity was proposed. Excessive accumulation of cholesterol plays a crucial role in the pathogenesis of various diseases (6). The integrity of the blood–brain barrier may be damaged by hypercholesterolemia (7). Very low-density lipoprotein (VLDL) cholesterol and its component apolipoprotein C3 (APOC3) promote inflammation and tissue damage by stimulating interleukin 1β (IL-1β), whereas high-density lipoprotein (HDL) cholesterol and its component apolipoprotein A1 (APOA1) decrease IL-1β release (8). Some studies indicate that APOA1 plays a role in the healing process of nerve and neuronal injury (9).

These results are valuable for basic and clinical studies of GBS but must be validated in as many studies as possible. We hypothesized that common lipid levels are associated with GBS. In this study, we performed an untargeted analysis in a case–control study to explore possible risk and protective factors associated with GBS. Remnant cholesterol and dyslipidemia were identified as potential risk factors for the onset and severity of GBS for the first time. In addition, we validated the effect of free cholesterol on monocyte activation at the cellular level.

## Methods

### Study Design and Participants

All patients with GBS were prospectively consecutively recruited at Beijing Tiantan Hospital, Capital Medical University from January 1, 2017, to December 31, 2021. The protocol was approved by the Ethics Committee of Beijing Tiantan Hospital (batch number: KY-2022-039-01). The data supporting the findings of this study can be obtained from the corresponding author upon reasonable request.

**Figure 1:**
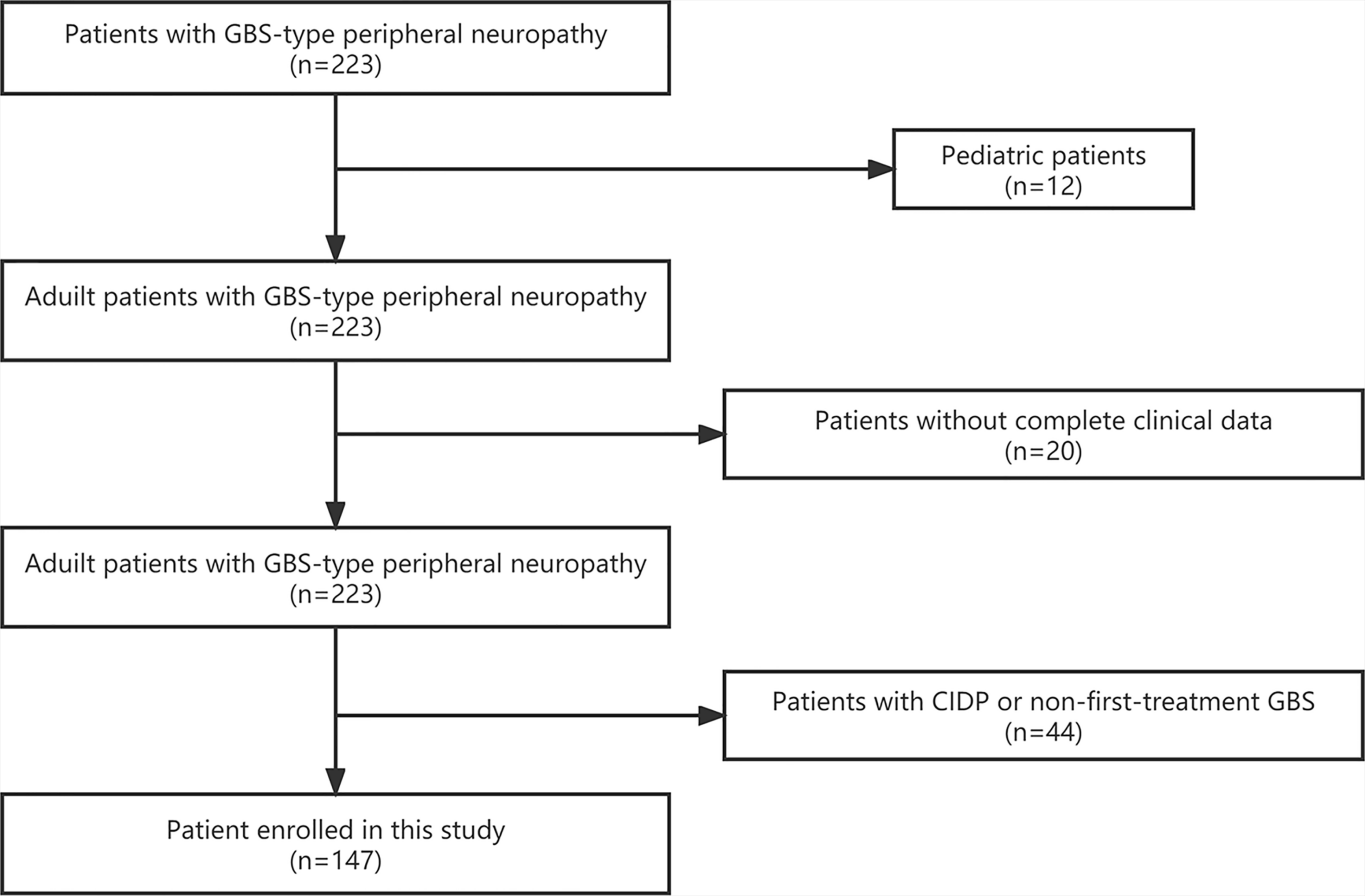
Flow diagram of the study participants.

GBS was diagnosed based on clinical symptoms, electrophysiological characteristics and cerebrospinal fluid characteristics, according to the guidelines published in 2014 (1): progressive weakness of limbs, including areflexia in weak limbs or decreased tendon reflexes (2); typical electromyography (3); albuminocytologic dissociation in cerebrospinal fluid analysis; and (4) absence of other possible neuropathic causes. From January 2017 to December 2021, 223 patients (including 221 adult patients) with GBS-type peripheral neuropathy were admitted to our hospital. Among the 221 adult patients, 22 patients without complete clinical data and 44 patients with chronic inflammatory demyelinating polyradiculoneuropathy or nonfirst-treatment for GBS were excluded. Ultimately, 147 (69.7%) of the 221 adult patients were included in this study (**Figure 1**). The onset date of GBS refers to the date of admission for symptoms diagnosed as GBS. Healthy individuals (matched by sex and age) from routine physical examinations were used as controls. According to their interviews and medical records, these individuals or their immediate family members did not have GBS or other demyelinating diseases.

### Data Collection

After resting in a seated position for 15 minutes, diastolic and systolic blood pressure in the right arm were measured with a standard mercury manometer. Peripheral blood samples were collected in the morning after fasting for 12 h. Body mass index (BMI) was calculated as weight (kg)/height (m2). Peripheral venous blood was used to measure the white blood cell count (WBC), lymphocyte count, monocyte count, albumin (ALB), immunoglobulin G (IgG), triglyceride, total cholesterol (CHO), HDL cholesterol, low density lipoprotein (LDL) cholesterol, APOA1, apolipoprotein B (APOB), and homocysteine (Hcy). Remnant cholesterol was calculated by subtracting HDL cholesterol and LDL cholesterol from CHO (10). Dyslipidemia was defined as at least one abnormality in CHO, triglyceride, HDL cholesterol, LDL cholesterol, APOA1 or APOB (11), and the dyslipidemia index was defined as the number of abnormalities for each of the six indicators.

Cerebrospinal fluid (CSF) samples were obtained by lumbar puncture. Lumbar puncture and peripheral blood collection were performed when the patient was in the acute phase and not receiving treatment. CSF samples were used to measure ALB, IgG, intrathecal immunoglobulin G synthesis rate of 24 h (24-h intrathecal IgG) and total protein.

The GBS disability score indicated the severity of disease. The GBS disability score at entry and at discharge were used to assess the functional status of patients with GBS (12, 13). The difference between the disability score at admission and discharge was defined as the disease remission score, which was used to evaluate the patient’s recovery. The evaluation criteria for health status are as follows: no symptoms, 0; mild symptoms and able to run, 1; can walk 10 m or more independently, but cannot run, 2; able to walk 10 m with help, 3; bedridden or wheelchair bound, 4; supplementary ventilation is required for at least part of the day, 5; or dead, 6. A severe condition was defined as a GBS disability score at admission of 3 or more, and fairly good condition was defined as a GBS disability score at admission of 2 or less (14). The Medical Research Council (MRC) sum score was used to assess muscle strength. The MRC sum score was calculated as the sum of MRC scores of 6 muscles of both upper and lower limbs, ranging from 60 (normal) to 0 (quadriplegic) (15).

The clinical features of GBS patients at admission included sex, age, preceding infection, symptoms of preceding infection, hypertension, smoking, alcohol consumption, diabetes, surgery, trauma, heart disease and hyperlipidemia.

### Preparation of PBMCs

Approximately 5 mL of peripheral blood was collected from 8 GBS patients and 8 healthy controls (from the above cohort and matched by sex and age) into EDTA-anticoagulated vacuum tubes. Peripheral blood mononuclear cells (PBMCs) were isolated using lymphocyte isolation agent (Sigma–Aldrich, USA).

### Cell Culture and Processing

The human monocyte leukemia cell line THP-1 was routinely maintained in RPMI 1640 (Invitrogen, USA) supplemented with 10% heat-inactivated (1 h at 56°C) FBS (AQ, China) and 1% β-mercaptoethanol (Invitrogen, USA) at 37°C in 5% CO2. THP-1 cells were cultured at a density of 5×105 cells/mL in 12-well culture dishes and treated for 24 h with 50 µg/mL free cholesterol (Sigma USA) or 100 ng/mL LPS in the presence or absence of 50 µg/mL free cholesterol.

### Quantitative Real-Time PCR (qRT–PCR)

Total RNA was extracted from PBMCs and THP-1 cells using TRIzol reagent (Invitrogen, USA) and reverse transcribed to cDNA using a Takara Prime Script RT Reagent Kit (Takara, Japan). qRT–PCR was conducted using a Roche Light Cycler 480 Real-Time PCR System (Roche, Switzerland) with a TB Green PCR Kit (Takara, Japan). The β-actin gene was used as the endogenous control. We used the 2-ΔΔCT method to calculate the relative gene expression. Primers for qRT–PCR are listed in **Table 1**.

**Table 1:** Clinical and Laboratory Characteristics in Patients With GBS and Healthy Controls .

	Controls	Patients with GBS		
Characteristics	N=153	N=147	t/z/x^2^	P value
Demographic				
Age,y	47.0 (31.5-60.0)	48.0 (33.0-60.0)	-0.535	0.592
Sex,male	85 (55.6)	82 (55.8)	0.002	0.968
Medical history, n (%)				
Hypertension	36 (23.5)	39 (26.5)	0.360	0.548
Diabetes mellitus	25 (16.3)	19 (12.9)	0.698	0.403
Heart disease	12 (7.8)	9 (6.1)	0.341	0.559
Cigarette smoking	25 (16.3)	41 (27.9)	6.487	0.011
Alcohol consumption	16 (10.5)	36 (24.5)	10.302	0.001
Surgery	10 (6.5)	36 (24.5)	18.615	< 0.001
Trauma	1 (0.7)	19 (12.9)	18.114	< 0.001
Hyperlipidemia	15 (9.8)	11 (7.5)	0.510	0.475
Preceding infection	28 (18.3)	91 (61.9)	59.561	< 0.001
Clinical features				
SBP, mmHg	119.86 ± 15.19	133.44 ± 17.32	-9.858	< 0.001
DBP, mmHg	72.42 ± 9.72	84.22 ± 11.00	-7.226	< 0.001
BMI, kg/m^2^	23.50 (20.85-25.60)	24.22 (21.48-26.45)	-2.104	0.035
Laboratory results				
Triglyceride, mmol/L	0.88 (0.63-1.21)	1.24 (0.88-1.93)	-6.838	< 0.001
Total cholesterol, mmol/L	4.41 ± 0.69	4.20 ± 0.87	2.315	0.021
HDL cholesterol, mmol/L	1.56 (1.37-1.78)	1.09 (0.93-1.25)	-11.175	< 0.001
LDL cholesterol, mmol/L	2.48 ± 0.66	2.55 ± 0.78	-0.831	0.406
Remnant cholesterol, mmol/L	0.36 (0.29-0.43)	0.49 (0.37-0.62)	-6.949	< 0.001
ApoA1, g/L	1.49 (1.35-1.63)	1.14 (1.02-1.28)	-10.902	< 0.001
ApoB, g/L	0.74 ± 0.17	0.89 ± 0.21	-6.779	< 0.001
Homocysteine, μmol/L	7.60 (6.79-8.41)	11.01 (8.70-12.69)	-10.450	< 0.001
Dyslipidemia index, n (%)			95.223	< 0.001
0	110 (71.9)	27 (18.4)		
1	20 (13.1)	44 (29.9)		
2	22 (14.4)	45 (30.6)		
≥3	1 (0.6)	31 (21.1)		

Data presented as mean ± SD, median (Q1-Q3) or percentage. DBP, diastolic blood pressure; SBP, systolic blood pressure; BMI, Body mass index; HDL, high-density lipoprotein; LDL, low-density lipoprotein; GBS, Guillain-Barré syndrome; APOA1, apolipoprotein A1; APOB, apolipoprotein B.

### Statistical Analysis

SPSS software (version 26.0) and GraphPad Prism software (version 8.0) were used for statistical analysis. Continuous data were analyzed using the independent t test or the Wilcoxon test. Categorical data were analyzed using the McNemar test. We performed a forward stepwise conditional logistic regression analysis to explore the independent risk factors for GBS and GBS with severe conditions. The Spearman rank correlation coefficient and Mantel–Haenszel test were performed to analyze the correlation between clinical characteristics and risk factor quartile.

## Results

### Clinical Characteristics of GBS Patients and Healthy Individuals

There were a total of 147 patients with GBS and 153 healthy controls consecutively included in our study. The characteristics of the GBS patients and univariate factors associated with the risk of GBS are presented in **Table 1**. The levels of diastolic blood pressure (DBP), systolic blood pressure (SBP), triglycerides, ApoB, and Hcy were higher in GBS patients than in healthy individuals (P<0.05 for all). In addition, patients with GBS have higher rates of smoking, alcohol consumption, surgery, trauma, hyperlipidemia, and preceding infection. Furthermore, CHO, HDL cholesterol and ApoA1 levels were lower in GBS patients. Dyslipidemia was found in 120 (1, 29.9%; 2, 30.6%; ≥3, 21.1%) patients with GBS and 43 (1, 13.1%; 2, 14.4%; ≥3, 21.1%) healthy controls (P<0.001).

### Potential Risk Factors for GBS

**Figure 2:**
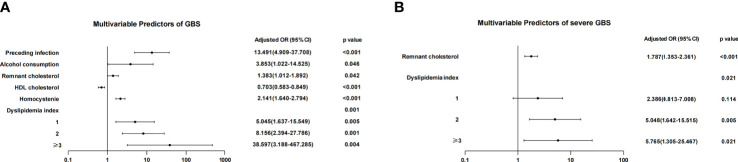
Logistic regression analysis of the risk of GBS and severe GBS. **(A)** After adjusting for multiple factors, preceding infection, alcohol consumption, remnant cholesterol, Hcy and the dyslipidemia index were independent risk factors for GBS. As the dyslipidemia index increased, the OR value increased. **(B)** Remnant cholesterol and a dyslipidemia index of 2 or ≥3 were independent risk factors for severe GBS.

The adjustment for potential covariates was made (**Figure 2**), including a history of smoking, surgery, trauma, SBP, DBP, BMI, triglycerides, CHO, APOB and APOA1. The logistic regression analyses showed that increased remnant cholesterol (odds ratio [OR], 1.383 [95% CI, 1.012–1.892]; P=0.042), Hcy (OR, 2.141 [95% CI, 1.640–2.794]; P<0.001) and dyslipidemia index (P=0.001) were correlated with the risk of GBS. The higher the dyslipidemia index, the higher the risk of GBS. In contrast, increased HDL cholesterol (OR, 0.703 [95% CI, 0.583-0.849]; P<0.001) was correlated with a lower risk of GBS.

### Analysis of Predictors for Severe GBS

Of 147 patients with GBS, 91 (61.9%) had severe GBS, and 56 (38.1%) had mild GBS. The univariate logistic regression analysis showed that SBP (OR, 1.021 [95% CI: 1.021]; P=0.042), triglycerides (OR, 1.128 [95% CI: 1.059-1.203]; P<0.001), HDL cholesterol (OR, 0.848 [95% CI: 0.747-0.963]; P=0.011), remnant cholesterol (OR, 1.886 [95% CI, 1.448-2.455]; P=0.042) and the dyslipidemia index (P<0.001) were correlated with severe GBS (**Table 2**). The multivariate logistic regression analysis showed that remnant cholesterol (OR, 1.787 [95% CI: 1.353-2.361]; P<0.001) and the dyslipidemia index (P=0.021) were significantly correlated with severe GBS (**Figure 2**).

**Table 2:** Univariate Logistic Regression Analysis of Predictors for Severe GBS.

Variables	Crude		
	OR	95%CI	P Value
Demographic			
Age,years	1.016	0.994-1.038	0.147
Sex,male	0.915	0.467-1.790	0.794
Medical history			
Hypertension	2.568	1.113-5.928	0.027
Diabetes mellitus	1.855	0.629-5.645	0.263
Heart disease	1.247	0.299-5.199	0.762
Cigarette smoking	1.470	0.685-3.155	0.323
Alcohol consumption	0.820	0.381-1.765	0.612
Surgery	1.313	0.596-2.895	0.499
Trauma	1.389	0.496-3.892	0.532
Hyperlipidemia	2.963	0.616-14.247	0.175
Symptoms of preceding infection	0.960	0.483-1.907	0.907
URTI	0.631	0.310-1.284	0.204
Diarrhea	1.395	0.649-3.003	0.394
Clinical features			
SBP, mmHg	1.021	1.001-1.042	0.042
DBP, mmHg	1.027	0.990-1.053	0.193
Body mass index, kg/m2	1.055	0.973-1.145	0.194
Laboratory results			
Triglyceride, mmol/L	1.128	1.059-1.203	< 0.001
Total cholesterol, mmol/L	1.237	0.837-1.827	0.287
HDL cholesterol, mmol/L	0.848	0.747-0.963	0.011
LDL cholesterol, mmol/L	1.108	0.718-1.708	0.644
Remnant cholesterol, mmol/L	1.886	1.448-2.455	< 0.001
ApoA1, g/L	0.780	0.660-0.922	0.004
ApoB, g/L	1.218	1.030-1.441	0.021
Homocysteine, μmol/L	1.009	0.963-1.058	0.702
Dyslipidemia index, n (%)			
1	2.850	2.050-16.670	0.001
2	5.846	2.050-16.670	0.001
≥3	16.031	4.214-60.982	< 0.001

DBP, diastolic blood pressure; URTI, SBP, systolic blood pressure; BMI, Body mass index; HDL, high-density lipoprotein; LDL, low-density lipoprotein; GBS, Guillain-Barré syndrome; APOA1, apolipoprotein A1; APOB, apolipoprotein B.

### Clinical Characteristics of GBS Patients Based on Remnant Cholesterol Quartiles and the Dyslipidemia Index

**Figure 3:**
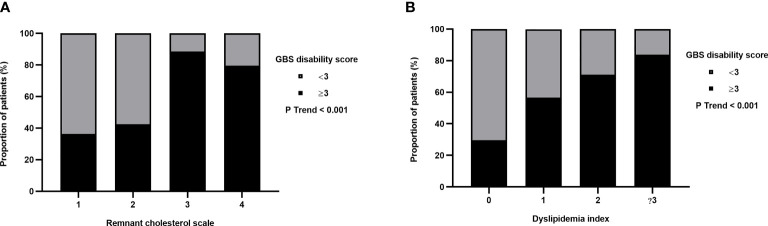
Proportional distribution of severe GBS. **(A)** Remnant cholesterol scale was set according to the quartile levels of the enrolled GBS patients. The proportion of severe GBS tended to increase with increasing remnant cholesterol levels. **(B)** The proportion of severe GBS tended to increase with an increase in the dyslipidemia index.

The clinical characteristics of GBS patients based on the remnant cholesterol quartiles are shown in **Table 3**. Remnant cholesterol quartiles were positively correlated with age, WBC count, monocyte count, GBS disability score at entry, GBS disability score at discharge and MRC sum score. The clinical characteristics of GBS patients based on the dyslipidemia index are shown in **Table 4**. The dyslipidemia index was positively correlated with the GBS disability score at entry and at discharge. Crucially, the proportion of severe GBS patients increased with the increase in the remnant cholesterol scale and dyslipidemia index (**Figure 3**).

**Table 3:** Characteristics of Patients with GBS According to Remnant Cholesterol Quartile.

	Remnant cholesterol, mmol/L					P Trend
Characteristics	All patients	Q1 (<0.37)	Q2 (0.37-0.49)	Q3 (0.49-0.62)	Q4 (>0.62)	
Patients	147	33	40	35	39	
Age, years	48.0 (33.0-60.0)	39.0 (25.0-60.0)	43.5 (29.3-61.0)	52.0 (36.0-60.0)	52.0 (44.0-60.0)	0.033
Sex, male	82 (55.8)	18 (54.5)	22 (55)	16 (45.7)	26 (66.7)	0.676
WBC count, 10^9^/L	6.38 ± 2.20	7.07 ± 2.29	8.25 ± 3.36	7.82 ± 2.36	7.82 ± 2.36	0.003
Lymphocyte count, 10^9^/L	1.69 ± 0.64	1.74 ± 0.59	1.96 ± 0.87	2.06 ± 1.08	2.06 ± 1.08	0.141
Monocyte count, 10^9^/L	0.40 ± 0.15	0.49 ± 0.19	0.54 ± 0.27	0.50 ± 0.22	0.50 ± 0.22	0.029
GBS disability score at entry						0.002
0 or 1	9 (6.1)	1 (3.0)	6 (15.0)	1 (2.9)	1 (2.6)	
2	47 (32.0)	20 (60.6)	17 (42.5)	3 (8.6)	7 (17.9)	
3	45 (30.6)	4 (12.1)	7 (17.5)	14 (40.0)	20 (51.3)	
4	40 (27.2)	6 (18.2)	9 (22.5)	17 (48.5)	8 (20.5)	
5	6 (4.1)	2 (6.0)	1 (2.5)	0 (0)	3 (7.7)	
GBS disability score at discharge						< 0.001
0 or 1	55 (37.4)	18 (54.5)	23 (57.5)	5 (14.3)	9 (23.1)	
2	40 (27.2)	8 (24.3)	10 (25.0)	10 (28.6)	12 (30.8)	
3	30 (20.4)	4 (12.1)	4 (10.0)	11 (31.4)	11 (28.2)	
4	18 (12.3)	2 (6.1)	2 (5.0)	9 (25.7)	5 (12.8)	
5	4 (2.7)	1 (3.0)	1 (2.5)	0 (0)	2 (5.1)	
Disease remission score						0.079
0	53 (36.1)	8 (24.2)	11 (27.5)	17 (48.6)	17 (43.6)	
1	75 (51.0)	21 (63.6)	22 (55.0)	13 (37.1)	19 (48.7)	
2	18 (12.2)	4 (12.1)	7 (17.5)	5 (14.3)	2 (5.1)	
3	1 (0.7)	0 (0)	0 (0)	0 (0)	1 (2.6)	
MRC sum score	44.90 ± 15.09	48.67 ± 11.55	46.95 ± 15.71	43.51 ± 13.42	40.85 ± 17.69	0.022
24-h intrathecal IgG	3.18 (-0.21-19.29)	2.24 (0.27-22.58)	6.75 (0.31-24.05)	3.94 (-9.08-17.24)	2.62 (-7.77-8.98)	0.188
CSF-ALB, mg/dl	0.37 (0.24-0.71)	0.35 (0.24-0.63)	0.39 (0.20-0.71)	0.43 (0.22-0.78)	0.33 (0.26-0.81)	0.448
Serum-ALB, mg/dl	38.37 ± 6.74	39.66 ± 6.18	39.93 ± 8.45	36.06 ± 4.68	37.37 ± 5.94	0.017
CSF-IgG, mg/ml	0.063 (0.036-0.183)	0.058 (0.035-0.149)	0.575 (0.28-0.160)	0.096 (0.039-0.25)	0.062 (0.038-0.215)	0.292
Serum-IgG, mg/ml	12.95 (10.40-24.53)	12.80 (11.30-19.80)	11.4 (10.23-16.70)	19.95 (11.70-31.20)	13.90 (9.88-25.85)	0.308
CSF-Pro, mg/dl	59.25 (38.80-110.64)	53.90 (38.35-89.55)	57.48 (31.45-110.81)	72.38 (36.24-134.07)	53.3 (39.34-133.52)	0.215
CSF-WBC,/ul	4.0 (2.0-7.5)	5.0 (2.0-9.0)	3.5 (2.0-6.75)	4.5 (2.0-5.25)	3.0 (2.0-7.5)	0.223
Albuminocytologic dissociation						0.522
Yes	62 (42.2)	15 (48.4)	14 (35)	13 (28.9)	20	
No	85 (57.8)	18 (52.6)	26 (65)	22 (71.1)	19	

WBC, white blood cell; CSF, cerebrospinal fluid; ALB, albumin; 24-h intrathecal IgG, intrathecal Immunoglobulin G synthesis rate of 24 hours;Pro, total protein.

**Table 4:** Characteristics of Patients with GBS According to Dyslipidemia Index.

	Dyslipidemia index					P Trend
Characteristics	All patients	0	1	2	≥3	
Patients	147	27	44	45	31	
Age, years	48.0 (33.0-60.0)	48.0 (29.0-65.0)	48.5 (29.3-60.8)	47.0 (35.0-61.5)	51.0 (40.0-59.0)	0.504
Sex, male	82 (55.8)	16 (59.3)	18 (40.9)	29 (64.4)	19 (61.3)	0.300
WBC count, 109/L	6.96 (5.63-8.69)	6.82 (4.70-7.86)	7.51 (6.15-8.91)	6.66 (5.14-8.57)	7.32 (6.42-9.21)	0.337
Lymphocyte count, 109/L	1.67 (1.26-2.31)	1.62 (1.51-1.99)	1.62 (1.05-2.24)	1.63 (1.25-2.27)	2.03 (1.50-2.68)	0.035
Monocyte count, 109/L	0.43 (0.34-0.56)	0.39 (0.32-0.60)	0.43 (0.34-0.54)	0.46 (0.34-0.64)	0.64 (0.36-0.54)	0.395
GBS disability score at entry						0.009
0 or 1	9 (6.1)	3 (11.1)	4 (9.1)	1 (2.2)	1 (3.2)	
2	47 (32.0)	16 (59.3)	15 (34.1)	12 (26.7)	4 (12.9)	
3	45 (30.6)	0 (0)	11 (25.0)	16 (35.6)	18 (58.1)	
4	40 (27.2)	8 (29.6)	13 (29.5)	11 (24.4)	8 (25.8)	
5	6 (4.1)	0 (0)	1 (2.3)	5 (11.1)	0 (0)	
GBS disability score at discharge						0.008
0 or 1	55 (37.4)	18 (66.7)	19 (43.2)	13 (28.9)	5 (16.1)	
2	40 (27.2)	4 (14.8)	10 (22.7)	14 (31.1)	12 (38.7)	
3	30 (20.4)	1 (3.7)	8 (18.2)	10 (22.2)	11 (35.5)	
4	18 (12.3)	4 (14.8)	7 (15.9)	4 (8.9)	3 (9.7)	
5	4 (2.7)	0 (0)	0 (0)	4 (8.9)	0 (0)	
Disease remission score						0.402
0	53 (36.1)	8 (29.6)	16 (36.4)	16 (35.6)	13 (42.0)	
1	75 (51.0)	15 (55.6)	22 (50.0)	22 (48.9)	16 (51.6)	
2	18 (12.2)	4 (14.8)	6 (13.6)	7 (15.5)	1 (3.2)	
3	1 (0.7)	0 (0)	0 (0)	0 (0)	1 (3.2)	
MRC sum score at entry	44.90 ± 15.09	48.85 ± 13.11	44.48 ± 14.89	43.36 ± 16.75	44.29 ± 14.56	0.164
24-h intrathecal IgG	3.18 (-0.21-19.29)	6.56 (0.14-25.74)	2.64 (-0.95-17.00)	3.00 (0.57-24.61)	4.55 (-1.67-12.69)	0.557
CSF-ALB, mg/dl	0.37 (0.24-0.71)	0.37 (0.25-0.74)	0.34 (0.18-0.71)	0.40 (0.24-0.69)	0.37 (0.25-0.73)	0.908
Serum-ALB, mg/dl	38.37 ± 6.74	38.63 ± 5.02	38.90 ± 8.19	37.49 ± 7.06	38.61 ± 5.63	0.653
CSF-IgG, mg/ml	0.063 (0.036-0.183)	0.070 (0.035-0.228)	0.058 (0.028-0.160)	0.073 (0.042-0.214)	0.065 (0.038-0.164)	0.979
Serum-IgG, mg/ml	12.95 (10.40-24.53)	12.45 (10.02-25.95)	11.8 (10.50-25.80)	14.50 (11.30-27.00)	12.55 (9.99-12.33)	0.865
CSF-Pro, mg/dl	59.25 (38.80-110.64)	57.56 (40.68-110.45)	60.38 (29.35-112.72)	60.23 (38.58-107.92)	56.46 (39.76-102.91)	0.882
CSF-WBC,/ul	4.0 (2.0-7.5)	3.0 (1.0-5.0)	4.0 (2.0-8.8)	4.0 (2.0-5.8)	5.0 (2.0-10.0)	0.211
Albuminocytologic dissociation						0.776
Yes	62 (42.2)	12 (44.4)	19 (43.2)	18 (40.0)	13 (41.9)	
No	85 (57.8)	15 (55.6)	25 (56.8)	27 (60.0)	18 (58.1)	

WBC, white blood cell; CSF, cerebrospinal fluid; ALB, albumin; 24-h intrathecal IgG, intrathecal Immunoglobulin G synthesis rate of 24 hours; Pro, total protein.

### Free Cholesterol Increases LPS-Induced THP-1 Cells

**Figure 4:**
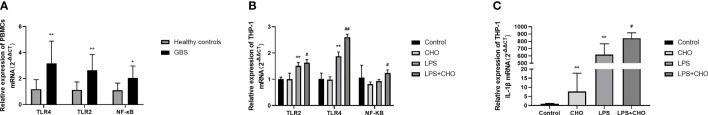
Free cholesterol promoted monocyte activation. **(A)** The mRNA levels of TLR2, TLR4 and NF-κB in the PBMCs of GBS patients were higher than those of healthy controls. *p<0.05, **p<0.01. **(B)** Compared with LPS stimulation alone, TLR2, TLR4 and NF-κB mRNA levels of THP-1 cells increased with free cholesterol and LPS costimulation. **: p < 0.01, compared to the control group; ^#^: p<0.05, compared to the LPS group; ^##^: P<0.01, compared to the LPS group. **(C)** Free cholesterol increased the mRNA expression of IL-1β in the presence or absence of LPS. **: p < 0.01, compared to the control group; #: p<0.05, compared to the LPS group.

Serum total cholesterol included conjugated cholesterol and free cholesterol. Depending on how remnant cholesterol was calculated, it mainly included free cholesterol and conjugated cholesterol from intermediate density lipoprotein cholesterol, VLDL cholesterol and chylomicrons. Free cholesterol is a key component of remnant cholesterol. To investigate the potential effect of remnant cholesterol on the activation of monocytes in PBMCs from patients with GBS, we measured the mRNA expression of TLR4, TLR2 and NF-κB in PBMCs and cultured cells. The results showed that the mRNA levels of TLR4, TLR2 and NF-κB associated with monocyte activation in the PBMCs of GBS patients were significantly higher than those of healthy controls (**Figure 4**). Moreover, THP-1 cells were treated with LPS, LPS/free cholesterol, and free cholesterol. Emerging experimental evidence has indicated that LPS activates THP-1 cells, and that free cholesterol treatment can increase the expression of TLR4, TLR2 and NF-κB mRNA in LPS-activated THP-1 cells (**Figure 4**). In addition, we found that free cholesterol increased the mRNA expression of IL-1β in the presence or absence of LPS (**Figure 4**).

## Discussion

GBS is the most common severe acute paralytic neuropathy, with a mortality rate of 5% and permanent sequelae rate of 10%. In the context of the COVID-19 pandemic, GBS is one of the serious sequelae (16, 17). It is of great importance to explore the risk factors for GBS disease and severe GBS. In our previous study on the proteomics differences between the CSF samples of GBS patients and the control group, it was found that the differential proteins were mainly enriched in lipid metabolism-related pathways (5). In this study, we explored the potential risk factors for GBS and severe GBS, taking serum lipid factors into account. The GBS patients included in this study reflected previously reported demographic and clinical characteristics (18). We found that preceding infection, alcohol consumption, increased remnant cholesterol, increased Hcy and an increased dyslipidemia index were correlated with a higher risk of GBS. In contrast, increased HDL cholesterol was associated with a lower risk of GBS. Furthermore, increased remnant cholesterol and an increased dyslipidemia index were correlated with a higher incidence of severe GBS. To explore the role of remnant cholesterol in the pathogenesis of GBS, we verified the function of free cholesterol in activating peripheral blood monocytes in patients with GBS. The mRNA levels of TLR4, TLR2 and NF-KB in the PMBCs of GBS patients were significantly higher than those of healthy controls, which is consistent with previous studies (19–21). Furthermore, free cholesterol significantly promoted monocyte activation induced by LPS and increased the mRNA expression of IL-1β.

The potential etiological factors of GBS were revealed in the results of our case–control study. Although many studies have been conducted on GBS, the cause of GBS is not fully understood. The majority of patients have had an event including preceding infection, vaccination and surgery in the four weeks before the onset of neurological symptoms of GBS (18, 22). GBS induced by preceding infections, particularly Campylobacter jejuni (C. jejuni), may be due to molecular mimicry between neural and microbial antigens. The glycans expressed on lipooligosaccharides (LOSs) of preceding infectious organisms were capable of inducing an antibody response to axolemmal surface molecules (23, 24). Campylobacter jejuni infection occurred frequently in the population, but acute motor axonal neuropathy after infection was rare. This was because ganglioside mimics were only present in a small percentage of C. jejuni strains, and there was a general immune tolerance to glycans on LOS in the population (25).

Our results suggest that alcohol consumption may be an independent risk factor for GBS. Repeated alcohol consumption can damage the central and peripheral nervous systems. The main consequences of long-term alcohol consumption are sleep disturbances, chronic pain, cognitive and behavioral disorders, stroke, and damage to peripheral nerves and muscles. The pathogenesis of alcoholic neuropathy remains controversial. It is currently primarily associated with nutritional deficiencies and the toxic effects of alcohol. Recent studies have suggested that alcohol consumption may lead to axonal degeneration resulting from oxidative stress, the release of proinflammatory cytokines and the activation of protein kinase C (26). Furthermore, alcohol-induced GBS has been reported in some cases (27, 28). These relationships make alcohol consumption a risk factor for GBS.

There is strong evidence that high Hcy may cause sensory and motor peripheral nerve dysfunction (29). Although it is unclear how increased Hcy leads to GBS, some explanations can be proposed. Low levels of vitamin B12 or folic acid can increase Hcy levels, and vitamin B12 deficiency can cause myelin damage due to inadequate methylation of myelin basic protein (30). Resulting from its special sensitivity to the nervous system, extracellular homocysteine may cause many harmful effects such as promoting excitatory toxicity by stimulating the n-methyl-D-aspartate receptor (NMDA) and damaging neuronal DNA to induce apoptosis (31). Hcy is considered an inflammatory marker, and Hcy itself can cause the breakdown of the blood–brain barrier (32). In this study, we demonstrated that increased Hcy in GBS patients significantly increases the risk of GBS. Moreover, many studies have shown that high Hcy levels are associated with the risk of multiple sclerosis, Alzheimer’s disease, skeletal muscle malfunction, and ocular diseases (33–36). Therefore, high homocysteine may be related to GBS.

Most importantly, our study suggested that various lipid levels were possible risk or protective factors for GBS. Compared with controls, patients with GBS had higher levels of triglycerides, LDL cholesterol, APOB and remnant cholesterol and lower HDL cholesterol and APOA1 levels. After considering the lipid levels as a dyslipidemia index, GBS patients had a higher dyslipidemia index. In addition, increased remnant cholesterol levels and an increased dyslipidemia index were associated with the incidence of severe GBS. Increased remnant cholesterol and dyslipidemia were associated with increased GBS disability scores. Furthermore, the proportion of patients with severe GBS increased with increasing remnant cholesterol and increasing dyslipidemia index. Some studies have shown a link between altered lipid metabolism and peripheral nerve dysfunction. The protective role of HDL cholesterol and apolipoproteins in established and early MS has been demonstrated. LDL cholesterol and serum total cholesterol were correlated with cognitive impairment in MS patients (37). In type 2 diabetes, dyslipidemia and hyperglycemia may damage dorsal root ganglion neurons and induce mitochondrial dysfunction (38). The risk of Alzheimer’s disease in old age was increased by elevated levels of LDL cholesterol in middle age (39). ApoA1 and HDL cholesterol play an important role in maintaining cerebrovascular integrity and reducing the risk of Alzheimer’s disease by reducing amyloid (Aβ) plaques and Aβ-mediated inflammation in the cerebrovascular system (40). In addition, various components of blood lipids may induce or inhibit systemic inflammation. VLDL cholesterol and its component APOC3 promote inflammation and tissue damage by stimulating IL-1β, whereas HDL cholesterol and its component apoA1 reduce IL-1β release (8). Immune dysfunction may be caused by excess cholesterol accumulation. Statins have been used in autoimmune diseases for decades with studies reporting beneficial effects on inflammatory and autoimmune diseases. This conclusion has been validated in multiple sclerosis, rheumatoid arthritis and systemic lupus erythematosus (6). Cholesterol crystals lead to activation of the NLRP3 inflammasome and lysosomal destruction and activate the complement pathway, both of which lead to subsequent caspase-1 activation and IL-1β release (41). Our results suggest that elevated levels of remnant cholesterol are associated with an increased number of white blood cells and monocytes in the blood. The mRNA levels of TLR2, TLR4 and NF-κB in PBMCs of GBS patients were higher than those of controls, which is consistent with previous reports. In addition, free cholesterol promoted LPS-induced monocyte activation and IL-1B release. These findings suggest that controlling lipid levels, particularly remnant cholesterol levels, may reduce the risk of GBS and severe GBS.

## Conclusions

In conclusion, preceding infection, alcohol consumption, remnant cholesterol, homocysteine and the dyslipidemia index were independent risk factors for GBS, and HDL-cholesterol was an independent protective factor for GBS. Furthermore, high remnant cholesterol levels and an elevated dyslipidemia index were associated with an increased risk of severe GBS. The mRNA levels of TLR2, TLR4, and NF-KB associated with monocyte activation were increased in GBS patients, and free cholesterol may promote this process.

## Limitations

There are several limitations in this study. First, because GBS is a rare disease, an insufficient number of cases were included in the study; this study was a case–control study. Second, this was a single-center study, which could lead to bias in the results due to regional differences. Finally, the effect of dyslipidemia on the pathogenesis of GBS should be demonstrated by additional cell experiments and animal experiments.

## Data Availability Statement

The raw data supporting the conclusions of this article will be made available by the authors, without undue reservation.

## Ethics Statement

The studies involving human participants were reviewed and approved by the institutional review boards of Beijing Tiantan Hospital. The patients/participants provided their written informed consent to participate in this study.

## Author Contributions

Study concept and design: GJZ. Acquisition of data: YD, LW, and JS. Analysis and interpretation of data: YD, LW, JS, YS, GL, and XL. Drafting of the manuscript: YD and LW. Statistical analysis: YD, LW, and JS. Critical revision of the manuscript for important intellectual content: GHZ and GJZ. All authors contributed to the article and approved the submitted version.

## Funding

This study was supported by Beijing Municipal Natural Science Foundation (code: NO.7222052), Beijing Hospitals Authority Clinical Medicine Development of Special Funding Support (code: ZYLX202108), Beijing Hospitals Authority’s Ascent Plan (DFL20220505) and Beijing High-level Public health technical Personnel Training program (2022-2-013).

## Conflict of Interest

The authors declare that the research was conducted in the absence of any commercial or financial relationships that could be construed as a potential conflict of interest.

## Publisher’s Note

All claims expressed in this article are solely those of the authors and do not necessarily represent those of their affiliated organizations, or those of the publisher, the editors and the reviewers. Any product that may be evaluated in this article, or claim that may be made by its manufacturer, is not guaranteed or endorsed by the publisher.

## Abbreviations

GBS: Guillain-Barré syndrome PNS: peripheral nervous system VLDL: Very low-density lipoprotein HDL: High density lipoprotein LDL: Low density lipoprotein APOA1: Apolipoprotein A1 APOB: Apolipoprotein B APOC3: Apolipoprotein C3 PBMCs: Peripheral blood mononuclear cells IL-1β: Interleukin 1β WBC: White blood cell count ALB: Albumin IgG: Immunoglobulin G CHO: Total cholesterol 24-h intrathecal IgG: Intrathecal Immunoglobulin G synthesis rate of 24 hours Hcy: Homocysteine BMI: Body mass index CSF: Cerebrospinal fluid MRC: Medical Research Council SBP: systolic blood pressure DBP: diastolic blood pressure THP-1: Human monocyte leukemia cell line TLR2: Toll-like receptor 2 TLR4: Toll-like receptor 4 NF-κB: Nuclear factor b OR: Odds ratio qRT-PCR: Quantitative Real-time PCR
